# Assessment of the performance of haematological and non-invasive fibrotic indices for the monitoring of chronic HBV infection: a pilot study in a Ghanaian population

**DOI:** 10.1186/s13104-023-06581-y

**Published:** 2023-11-04

**Authors:** Eric N. Y. Nyarko, Christian Obirikorang, W. K. B. A. Owiredu, Evans Asamoah Adu, Emmanuel Acheampong

**Affiliations:** 1 Department of Chemical Pathology, University of Ghana Medical School, University of Ghana, Accra, Ghana; 2 Department of Molecular Medicine, School of Medicine and Dentistry, Kwame Nkrumah University of Science and Technology, Kumasi, Ghana; 3 School of Medical and Health Sciences, Edith Cowan University, Joondalup, Australia; 4 Global Health and Infectious Diseases Group, Kumasi Centre for Collaborative Research, Kumasi, Ghana

**Keywords:** Hematological, Biochemical, Fibrotic indices, Ghanaians, HBV infection, Chronic HBV status

## Abstract

**Objective:**

Haematological and liver fibrotic markers could be appreciably utilized for effective monitoring of Chronic Hepatitis B viral (HBV) infection, thereby increasing patient’s treatment outcome. The objective of this study was to assess the applicability of complete blood count (CBC) and non-invasive liver-fibrotic indices as markers of prognostic outcome and monitoring in HBV infections.

**Results:**

Significant differences in levels of white cell and differentials counts, red blood cell count, hemoglobin indices, and platelet indices were observed between HBV-infected patients (cases) and uninfected persons (controls). Levels of haemoglobin (Hb), total white blood cells (tWBC), neutrophils, monocytes, platelets, and Platelet Distribution width (PDW) were significantly lower (p < 0.05) in the cases compared to the controls. Total and indirect bilirubin; De-Ritis ratio, Aspartate transaminase to platelet ratio index (APRI) and RDW-to-platelet ratio (RPR) were elevated in cases compared with controls (p-value < 0.05). In a multivariate adjusted model to test the significance of markers, Hemoglobin Index (beta coefficient = − 0.876, p-value < 0.001), NLR (beta coefficient = − 0.839, p-value < 0.001), MPV_10000 (beta coefficient = − 0.333, p-value < 0.001) and Albumin (beta coefficient = − 0.059, p-value = 0.014), were associated with HBV infection status. Receiver operative characteristics curve analysis showed Hemoglobin Index (AUC = 0.744) and MPV_10000 (AUC = 0.730) as better prognostic markers for HBV-infection.

**Supplementary Information:**

The online version contains supplementary material available at 10.1186/s13104-023-06581-y.

## Introduction

Africa is a home of two-third of the 257 million HBV infected individuals [1]. The national prevalence of HBV infection in Ghana is average 12.3% [2]. According to this, every 10th Ghanaian is infected with HBV, putting them at risk of end stage liver complications such as cirrhosis and liver cancer [3, 4], after a period of chronicity. Consequently, early detection, treatment and frequent monitoring (especially among persons chronically infected) are critical components of comprehensive management of HBV infected persons. Physical examination and the use of both traditional and novel immunological, biochemical, and haematological markers are used to monitor HBV infections. There are reports on the use of immunological markers for screening, diagnosis and monitoring, including HBV profiling of viral antigens and host antibodies [4–6], however there is paucity of data with regards to the use of haematological and fibrotic indices for the monitoring of HBV, espeacially Chronic HBV infections. Chronic HBV (CHBV) infection is the persistence of hepatitis B surface antigen (HBsAg) for 6 months or more in serum of an infected person, after acute infection with HBV. The progression and outcome of CHBV infection is dependent on viral pathogenicity, host immune status and host-virus interaction [7]. It is characterized by wide biochemical, and immunological profiles, which differs from one individual to another with regards to ALT and AST activities, presence or absence of HBeAg, and hepatic fibrosis—throughout each of the 4 phases of CHBV infection [8]. The most commonly used tests for HBV monitoring are the liver function biochemical tests (LFT’s) which include measuring of the concentrations of serum bilirubin’s and some proteins as well as activity of enzymes like alanine transaminase (ALT), aspartate transaminase (AST), alkaline transaminase (ALP) and gamma-glutamyl transferase (GGT) [9]. However, HBV affects other parameters, such as haematological indices, in diverse ways. For instance, liver inflammation caused by HBV infection leads to the release of hepcidin which inhibits iron absorption and recycling, resulting in hypoferremia, and consequently restricted erythropoiesis and low Hb levels [10]. HBV also causes reduced total white blood cell (tWBC) count by inhibiting the erythropoietic function of the bone marrow, while conversely, causing elevated lymphocyte levels due to concerted efforts by the immune system to eliminate HBV and infected cells in order to protect the entire liver [11–13]. Beside these, another important haeamatological parameter affected by HBV infection is the platelets. Platelet production is partly ‘controlled’ by thrombopoietin, a hormone made by the kidneys and largely by the liver. The inflammation process in the liver by the HBV reduces thrombopoietin synthesis by the liver, and subsequently affects the levels of thrombocytes produced by the bone marrow. In chronic HBV infections, the stage of liver fibrosis is important for clinical assessment and management. Although the gold standard has been the liver biopsy for histological investigations, it is an invasive and expensive procedure, not suitable for resource limited settings and its very subjective, making its accuracy sometimes questionable. Moreover, data on the use of haematological indices and non-invasive liver fibrotic tools for screening, prognosis and monitoring purposes are few, particularly in Ghana—a country with high HBV infection rate and limited laboratory resources. Hence this pilot study seeks to bridge the knowledge gap about the levels of haematologic indices among HBV infected individuals and their application in monitoring HBV infection, especially CHBV, as well as to assess four (4) non-invasive fibrotic indices among Ghanaians with HBV infection.

## Methods

There were 300 participants in this case–control study, with 150 HBV infected individuals as cases and 150 apparently healthy HBV non-infected individuals as controls. 89 of the cases with persistence of the HBsAg in the blood for more than 6 months were considered to have chronic HBV (CHBV) infection, and the rest (61) acute HBV infection. The study was ethically approved by Committee of Human Research, Publication and Ethics (CHRPE) of the Kwame Nkrumah University of Science and Technology, (KNUST), Kumasi–Ghana (Refs: CHRPE/RC/053/18, CHRPE/AP/426/19). Written informed consent were obtained from the participants. The sample size was calculated as described by Charan & Biswas [14]. Six milliliters (6 ml) of venous blood sample were collected from each participant—3 ml was put into serum separator gel tube (SST) and 3 ml into EDTA- anticoagulated tube. The SST sample was allowed to clot and centrifuged at 2500 g for 7 min to obtain serum and preserved below − 20 ℃ till assayed.

Different biochemical and hematological procedures and techniques were employed, depending on the target analyte. Haemoglobin, blood cells and other haematological indices were measured using the vis-spectrophotometry, impedance, and flow cytometry principles, with Mindray 5-parts hematology analyzer (Mindray, Shenzhen-China, 2013), while the HumaStar 200 Clinical chemistry analyzer (Human Diagnostics, Germany, 2014) was used to measure the concentrations of the bilirubins (total and direct), albumin and total protein. The activity of each of the enzymes: ALP, ALT, GGT and AST were also measured.

Data were entered into the Statistical Package for the Social Sciences (SPSS, version 23). The data were coded and analyzed. The categorical data were presented by the frequencies of their occurrences. Chi-Square test was used to make comparisons among the categorical groups. Normality of continuous data was checked using Gaussian distribution. De-Ritis ratio was calculated by dividing AST by ALT, APRI by this formulae $\{[\mathrm{AST\,level }(\mathrm{IU}/\mathrm{L})/\mathrm{ AST }(\mathrm{ULN\,in\,IU}/\mathrm{L})]/[\mathrm{Platelet\,count }(109 )]\},$ FIB-4 index =$[Age (yrs) x AST (IU/L)] / [Platelet\,count x \surd ALT (IU/L)]$ and RPR = $\mathrm{RDW }(\mathrm{\%}) /\mathrm{ Platelet\,count }(10^9//\mathrm{L})$. The biochemical, haematological, and fibrotic indices were presented as mean ± standard deviation and the T-test was used to compare two groups, while ANOVA was used to compare the three groups (i.e. Controls, Acute HBV participants, and Chronic HBV Participants). Statistical significance was defined by P-value of less than 0.05. Principal component analysis was undertaken to create uncorrelated components to maximize variance among highly correlated variables. Overall, a haemoglobin index (consisting of Hb, Hct and MCH) was generated. Also, because neutrophils count strongly negatively correlated with lymphocyte counts, we generated a neutrophil-to-lymphocyte ratio (NLR), which has been previously validated to represents systemic inflammation marker [15–17]. A stepwise (forward conditional) multivariate linear regression analysis, adjusting for age and BMI was used to evaluate a linear association of routine markers with CHB (Table 2). Significant markers were used in a multivariate logistic regression analysis. Receiver operative characteristics curve analysis was performed to examine the performance of significant markers for CHB prognosis.

## Results

There was no significant difference between the number of male participants and the number of female participants (p > 0.05). Participants within the age bracket 18–36 years were the mode (76.7%) (Table 1). In the comparison of the haematological indices between cases and controls, apart from RBC count, MCV, PCT and haematocrit level (p-value > 0.05), cases and control participants significantly differed in terms of all the haematological parameters (Table 1).

**Table 1:** Sociodemographic, Haematological and Fibrotic indices of the Participants

Variable	Category	Cases (n = 150) mean (SD)	Controls (N = 150) mean (SD)	P-value
Gender	Male	59 (39.3)	69 (46.0)	*0.24*
	Female	91 (60.7)	81 (54.0)	
Age (Yrs.)^#^	18–36	116 (79.5)	111 (74.0)	*0.05*
	37–53	30 (20.5)	33 (22.0)	
	54–64	0 (0.0)	6 (4.0)	
Hb (12.0–18.0) (g/dl)		12.3 (2.1)	12.9 (1.9)	*0.01*
Total WBC count (4.3–11.5) 10^9^/L		5.9 (1.8)	6.8 (3.1)	< *0.001*
Neutrophils (50.0–70.0%)		45.9 (13.4)	57.6 (15.3)	< *0.001*
Lymphocytes (20.0–40.0%)		44.1 (13.0)	32.8 (14.2)	< *0.001*
Monocytes (3.0–12.0%)		5.8 (4.8)	6.9 (2.7)	*0.01*
Eosinophils (0.5–5.0%)		3.2 (3.1)	2.5 (2.8)	*0.03*
Basophils (0.0–1.0%)		1.2 (1.6)	0.3 (0.6)	< *0.001*
Platelets (100–300) × 10^9^/L		195 (53)	222 (70)	< *0.001*
Hematocrit (37.0–54.0%)		*36.7 (6.7)*	*35.4 (5.9)*	0.09
RBC's (3.5–5.5) × 10^12^/L)		*4.3 (0.7)*	*4.2 (0.6)*	0.13
MCV (80.0–100.0fL)		*83.2 (11.1)*	*83.9 (7.5)*	0.47
MCH (27.0–34.0 pg)		*28.3 (5.5)*	*30.9 (5.7)*	< *0.001*
MCHC (32.0–36.0 g/dL)		*32.9 (3.2)*	*36.2 (3.0)*	< *0.001*
RDW-CV (11.0–16.0%)		*15.3 (4.4)*	*13.4 (1.4)*	< *0.001*
RDW-SD (35.0–56.0fL)		*46.7 (7.8)*	*42.8 (5.4)*	< *0.001*
PDW (9–17)		*14.5 (3.2)*	*15.9 (2.3)*	< *0.001*
PCT (0.20–0.35%)		*0.23 (0.07)*	*0.22 (0.07)*	< *0.001*
MPV (8.0–11.5fL)		*12.2 (4.3)*	*9.8 (1.3)*	< *0.001*
De-Ritis ratio		*1.350* ± *1.268*	*1.094* ± *0.410*	*0.02*
FIB-4		*1.082* ± *1.122*	*0.867* ± *0.658*	*0.05*
APRI		*0.431* ± *0.335*	*0.349* ± *0.268*	*0.01*

Data is presented as mean ± SD

*p*-values < 0.05 were considered statistically significant

*Hb* hemoglobin, *WBC* White Blood Cell, *RBC’s* Red Blood Cells, *MCV* Mean Cell Volume, *MCH* Mean Cell hemoglobin, *MCHC* Mean Cell hemoglobin Concentration, *RDW-CV* Red cell Distribution Width -Coefficient of Variation, *RDW-SD* Red cell Distribution width—Standard Deviation, *PDW* Platelet Distribution width, *PCT* Plateletcrit and *MPV* Mean Platelet Volume, *APRI* Aspartate aminotransferase-to-platelet ratio index, *FIB-4* fibrosis 4 index, *RPR* RDW-to-platelet ratio

^#^4 Participants did not provide their ages

The bilirubin (total and indirect, but not direct) was significantly higher in the case group compared to the control group (p < 0.01). However, albumin level was significantly lower in cases compared with controls (39.78 vs 41.58, p-value = 0.017). The levels of de -Ritis ratio, APRI and RPR were statistically higher in the cases (p < 0.05) (Table 1).

The output of the regression model is shown in Additional file 1: Table S1. Heamoglobin index (Hb-index), MPV_10000, NLR, albumin, AST, GGT, PDW, and FIB-4 had a significant linear association with CHB. Thus, were used in a multivariate logistic regression analysis (Table 2). Overall, haemoglobin index, NLR, and Albumin were significantly reduced in CHB infected individuals, whereas MPV_1000 was significantly increased, compared to the controls.

**Table 2:** Stepwise Regression model to examine an association between CHB infection and routine laboratory markers

Model	Predictors	Beta coefficient	Exp(B)	95% C.I. for EXP(B)		Sig
				Lower	Upper	
Step 1a	Haemoglobin Index	− 0.92	0.398	0.299	0.531	< 0.0001
Step 2b	Haemoglobin Index	− 0.879	0.415	0.306	0.563	< 0.0001
	MPV_10000	0.332	1.393	1.221	1.59	< 0.0001
Step 3c	Haemoglobin Index	− 0.89	0.41	0.296	0.57	< 0.0001
	NLR	− 0.821	0.44	0.313	0.618	< 0.0001
	MPV_10000	0.312	1.366	1.193	1.565	< 0.0001
Step 4d	Haemoglobin Index	− 0.876	0.416	0.298	0.581	< 0.0001
	NLR	− 0.839	0.432	0.305	0.613	< 0.0001
	MPV_10000	0.333	1.395	1.212	1.606	< 0.0001
	Albumin	− 0.059	0.943	0.9	0.988	0.014

Association of CHB infection with routine laboratory markers

*MPV* Mean Platelet Volume, *NLR* Neutrophil-to-lymphocyte ratio

**Fig. 1:**
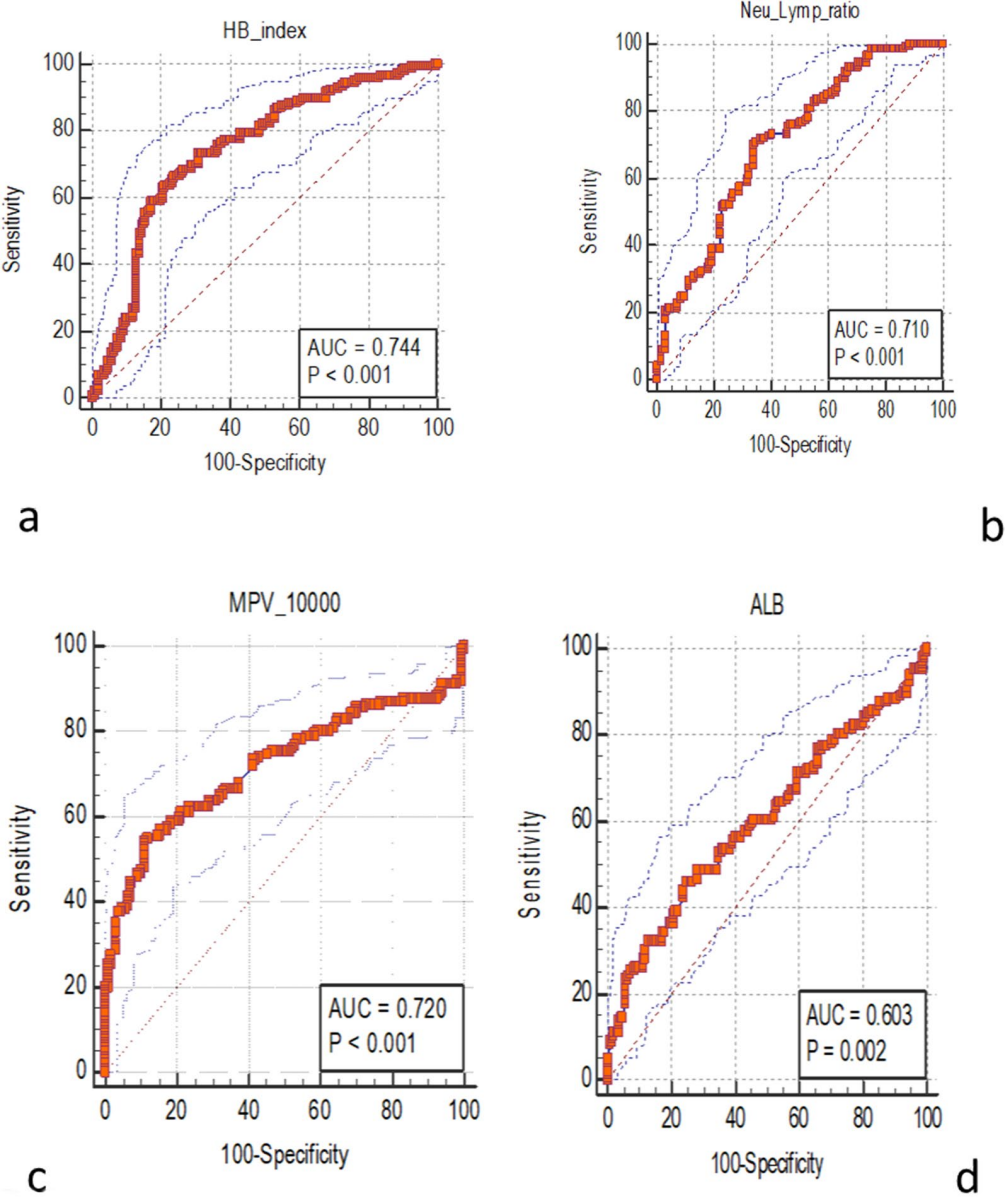
Receiver Operative Characteristics (ROC) curve analysis of routine markers associated with CHB infections. **a**: Haemoglobin (Hb) index, **b**: Neutrophil–lymphocyte-ratio (NLR), **c**: MPV_10000, **d**:Albumin

The ROC curved analysis found Hb-index, MPV-1000 and Neu-to-Lym ratio (NLR) as the best prognostic markers of CHB, in the order of significance (Fig. 1).

The study further compared the levels of the haematological and the fibrotic indices between the controls, cases with acute HBV infection and cases with chronic HBV infection (Table 3). The comparison found some significant differences in both the haematological and fibrotic indices; however, those differences were stronger and mostly between the acute infection group and the controls or the chronic infection group and the controls.

**Table 3:** Comparison of Haematological and Fibrotic indices between the Controls and HBV infected categories

Haematological index	Control (n = 150)	Acute HBV patients (n = 61) mean (SD)	Chronic HBV patients (N = 89) mean (SD)	P-value
Hb (12.0–18.0) (g/dl)	12.9 (1.9)	12.3 (2.1)	12.2 (2.1)	*0.021*
Total WBC count (4.3–11.5) 10^9^/L	6.9 (3.1)	5.9 (1.8)	5.8 (1.9)	*0.003*
Neutrophils (50.0–70.0%)	57.4 (15.4)	45.8 (13.5)	46.1 (13.6)	< *0.001*
Lymphocytes (20.0–40.0%)	33.0 (14.2)	46.1 (13.3)	42.8 (12.9)	< *0.001*
Monocytes (3.0–12.0%)	6.9 (2.7)	4.6 (2.7)	6.5 (5.6)	*0.001*
Eosinophils (0.5–5.0%)	2.5 (2.8)	2.5 (1.6)	3.7 (1.5)	*0.010*
Basophils (0.0–1.0%)	0.3 (0.6)	0.9 (0.7)	1.4 (1.8)	< *0.001*
Platelets (100–300) × 10^9^/L	222 (70)	192(46)	188 (58)	*0.002*
Hematocrit (37.0–54.0%)	*35.4 (5.9)*	37.5 (6.9)	37.2 (6.2)	0.076
RBC's (3.5–5.5) × 10^12^/L)	*4.2 (0.6)*	4.3 (0.7)	4.3 (0.7)	0.140
MCV (80.0–100.0fL)	*83.9 (7.5)*	86.4 (11.7)	85.7 (10.1)	0.490
MCH (27.0–34.0 pg)	*30.9 (5.7)*	28.3 (5.9)	28.1 (5.0)	*0.010*
MCHC (32.0–36.0 g/dL)	*36.2 (3.0)*	32.8 (7.0)	32.8 (5.2)	*0.026*
RDW-CV (11.0–16.0%)	*13.4 (1.4)*	13.5 (1.6)	12.8 (1.3)	< *0.010*
RDW-SD (35.0–56.0fL)	*42.8 (5.4)*	*48.8 (9.1)*	*38.7 (7.0)*	< *0.010*
PDW (9–17)	*15.9 (2.3)*	*14.7 (3.4)*	*14.9 (2.5)*	< *0.010*
PCT (0.20–0.35%)	*0.22 (0.07)*	*0.25 (0.10)*	*0.26 (0.07)*	< *0.001*
MPV (8.0–11.5fL)	*9.8 (1.3)*	*13.2 (7.3)*	*12.2 (4.4)*	< *0.001*
De-Ritis ratio	*1.01* ± *0.41*	*1.09* ± *1.27*	*1.35* ± *0.84*	*0.038*
FIB-4	*0.87* ± *0.66*	*1.07* ± *1.12*	*1.16* ± *1.03*	*0.032*
APRI	*0.350* ± *0.27*	*0.39* ± *0.34*	*0.38* ± *0.29*	*0.010*
RPR	*0.06* ± *0.04*	*0.07* ± *0.05*	*0.08* ± *0.05*	< *0.001*

Data is presented as mean ± SD

*p*-values < 0.05 were considered statistically significant

*Hb* hemoglobin, *WBC* White Blood Cell, *RBC’s* Red Blood Cells, *MCV* Mean Cell Volume, *MCH* Mean Cell hemoglobin, *MCHC* Mean Cell hemoglobin Concentration, *RDW-CV* Red cell Distribution Width -Coefficient of Variation, *RDW-SD* Red cell Distribution width—Standard Deviation, *PDW* Platelet Distribution width, *PCT* Plateletcrit and *MPV* Mean Platelet Volume, *APRI* Aspartate aminotransferase-to-platelet ratio index, *FIB-4* fibrosis 4 index, *RPR* RDW-to-platelet ratio

## Discussions

This study aimed to find out if hematological, biochemical, and liver fibrotic indices in Ghanaians with chronic hepatitis B (CHB) infection could be helpful, reliable, and inexpensive markers for HBV infection monitoring and evaluation. Hematological markers, non-invasive fibrotic indices, as well as liver function tests, were found to be beneficial in the monitoring of Chronic HBV (CHBV) infection. In CHB infection, both liver enzymes and blood cell indices were significantly different from normal controls. The study observed that the total white blood cell (TWBC) count, neutrophils, and monocytes in the Chronic HBV infected patients were significantly lower compared with that of the controls. According to Sing et al. [18], HBV suppresses the erythropoietic activity of the bone, lowering the total cell count. That is, the virus frequently suppresses haematopoiesis and myelopoiesis reducing the number of neutrophils produced. In addition, HBV has been shown to destroy neutrophils which generally make up > 55% of the TWBC count, and the virus’s HBsAg has been identified in the nucleus juvenile neutrophils, which is thought to inhibit and decrease neutrophil development [19]. Moreover, Li et al. [20], found considerably higher amounts of basophils, eosinophils, lymphocytes, and reduced neutrophils in HBV infected persons than the controls. Asaduzzaman, et al. [21] made a similar finding in chronic HBV patients, where higher eosinophil levels were found. Other chronic HBV investigations have also observed increased production and peripheral blood levels of lymphocytes such as CD64 and effector CD8 + T cells [11, 12]. The increase in lymphocytes is most likely the immune system’s coordinated attempts to remove HBV infected hepatocytes, to protect the entire liver. We found the case group had significantly lower haemoglobin levels than the control group in this study. Similar to our finding, Francisca et al. [13], observed that HBV infected patients have decreased Hb levels compared with HBV non-infected parsons. The low Hb levels in the HBV infected persons could be due to increased production and secretion of hepcidin—a molecule useful in the metabolism of iron- in uncomplicated chronic HBV infection [10]. Furthermore, since globin (the major protein in albumin) production is required for haemoglobin synthesis, the lower levels seen in the case group could explain our findings.

In the HBV infected cases, we observed significantly lower MCH and MCHC compared with the controls. Mao et al. [22], reported abnormal iron metabolism as a feature in CHBV infection. Also, transferrin and ferritin production are compromised in CHB infections. Accordingly, iron deficiency anaemia characterized by low MCH and MCHC is observed in some cases of CHB infections. The study observed that platelets and PDW were significantly decreased in cases compared to the control group. However, MPV was significantly elevated in the case group compared with the control group. Other studies have reported reduced platelet count and PDW among HBV infected persons [23, 24]. Also in a number of studies, elevated MPV has been reported among chronic HBV patients [24, 25], which is in agreement to this present findings. In a study by Pan et al. [23], reduced thrombopoietin production was observed in inflamed liver cells due to HBV infection. It has also been reported that in HBV infected persons, hypersplenism as a result of portal hypertension may lead to increased sequestration of blood cells including platelets. Also, HBV fibrotic process is characterized by transport of thrombocytes to the liver and its vessels thereby reducing the platelets circulating in the blood. The significance of these haematological indices are affirmed by the findings that haemoglobin index (Hb-index) and NLR were significantly reduced in CHB infected individuals whereas MPV_10000 was significantly increased among same, compared with controls. Besides, all three (3) parameters were found to be possible prognostic and monitoring markers of chronic HBV infection. Similar fi have been reported by Liu et al. [26].

In chronic HBV infections, the stage of liver fibrosis is important for clinical assessment and management. That is, the treatment and prognosis of CHB infection largely depends on the fibrosis stage. Although the gold standard procedure has been the liver biopsy for histological examination, it is an invasive and expensive procedure, not suitable for resource limited settings and its very subjective, making its accuracy sometimes questionable. Thus, we compared the values of de-Ritis ratio, APRI, FIB-4 and RPR indices in HBV infected individuals and healthy controls. Subsequently, we compared the values of these liver fibrosis indices between the CHBV patients, the acute HBV patients, and the control groups. We observed that the de-Ritis ratio, APRI and RPR fibrotic indicators were significantly elevated in cases (-to a greater extent in CHB patients than the acute group), whiles some key liver enzymes (ALT, AST, GGT and ALP) could not differentiate between cases and healthy controls. This shows some benefits of fibrotic indices to LFTs in monitoring HBV associated liver fibrosis, common (but very mild and slow) in inactive CHB. Similar studies by Song and Kim [8], Liu et al. [26] and Sebastiani et al. [27], reported that APRI is one of the accurate and reliable early non-invasive index for detecting hepatic fibrosis in CHB infection. Regarding the RPR, low circulating platelets [28] and high RDW are associated with severity of hepatic fibrosis or cirrhosis [29, 30] which was a feature observed among the CHB cases in this study. Thus, evaluation of all these calculators as predictors of HBV -associated fibrosis and cirrhosis among Ghanaians would be very useful.

The outcomes of this study show that there is a significant shift in WBC and its differentials (including NLR), platelet and platelet derived indices, RBC indices in Chronic HBV (CHB) infection. However, these haematological indices are susceptible to many infections, inflammations and other medical conditions, and cannot be used in isolation for monitoring and evaluation of chronic or acute HBV infections. APRI, de-Ririts ratio and RPR were also found to be better indicators to discriminate CHB infected individuals from healthy patients, and those with acute infection. Further studies needed to assess the diagnostic ability of these indices among the general Ghanaian population as well as focus on HBV infected subgroups and phases are recommended.

### Limitations of the study

Though we have demonstrated the usefulness of these haematological and non-invasive fibrotic indices for CHBV infection monitoring, with some congruence with previous reports, this study is not without limitations:

- 1.The study was localized within the Greater Accra region of Ghana, and generalization of the findings (to other regions within the country) should be done with caution.
- 2.Also, the sample size of the study was small, which may have affected the power.

Thus, further studies, with larger sample sizes across the other all 16 regions are recommended.

### Supplementary Information

**Additional file 1: Table S1.** Regression Model. **Table S2.** Biochemical Analytes of the Study of the study participants.

## Data Availability

Two Supplementary tables (Additional file 1: Tables S1, S2) are attached to this publication. All other datasets used and/or analysed during this study may be obtained from the corresponding author upon reasonable request.

## Abbreviations

CHBV: Chronic Hepatitis B Viral infection
HBV: Hepatitis B virus
LFT’s: Liver function tests
ALT: Alanine transaminase
AST: Aspartate transaminase
GGT: Gamma-glutamyl transferase
ALP: Alkaline phosphate
HBsAg: Hepatitis B surface antigen
HBsAb: Hepatitis B surface antibody
HBeAg: Hepatitis B envelop antigen
HBeAb: Hepatitis B envelop antibody
HBcAb: Hepatitis B core antibody
Hb: Hemoglobin
WBC: White blood cell
RBC's: Red blood cells
MCV: Mean cell volume
MCH: Mean cell hemoglobin
MCHC: Mean cell hemoglobin concentration
RDW-CV: Red cell distribution width-coefficient of variation
RDW-SD: Red cell distribution width—standard deviation
PDW: Platelet distribution width
PCT: Plateletcrit
MPV: Mean platelet volume
APRI: Aspartate aminotransferase-to-platelet ratio index
FIB-4: Fibrosis index
RPR: RDW-to-platelet ratio
NLR: Neutrophil-to-lymphocyte ratio
