# Supplementary material for: Assessment of the performance of haematological and non-invasive fibrotic indices for the monitoring of chronic HBV infection: a pilot study in a Ghanaian population
Source: BMC Res Notes. 2023 Nov 4;16:312. doi: 10.1186/s13104-023-06581-y (PMC10625242; doi:10.1186/s13104-023-06581-y)
Supplement: Supplementary file 1 — Additional file 1: Table S1. Regression Model. Table S2. Biochemical Analytes of the Study of the study participants. [file 13104_2023_6581_MOESM1_ESM.docx]

**Additional file Data**

**Table S1. Regression Model**

| **Model** | **Model Parameters** | **Standardized Beta Coefficients** | **T** | **Sig.** | **95.0% Confidence Interval for B** | |
| --- | --- | --- | --- | --- | --- | --- |
|  |  |  |  |  | **Lower Bound** | **Upper Bound** |
| 1 | (Constant) |  | 1.14 | 0.256 | -0.24 | 0.88 |
|  | AGE | -0.03 | -0.52 | 0.607 | -0.01 | 0.01 |
|  | BMI | 0.05 | 0.85 | 0.397 | -0.01 | 0.03 |
| 2 | (Constant) |  | 1.61 | 0.109 | -0.09 | 0.93 |
|  | AGE | 0.02 | 0.40 | 0.687 | -0.01 | 0.01 |
|  | BMI | 0.01 | 0.14 | 0.889 | -0.02 | 0.02 |
|  | Haemoglobin index | -0.41 | -7.36 | 0.000 | -0.26 | -0.15 |
| 3 | (Constant) |  | 1.68 | 0.094 | -0.07 | 0.93 |
|  | AGE | 0.04 | 0.69 | 0.489 | 0.00 | 0.01 |
|  | BMI | 0.04 | 0.71 | 0.479 | -0.01 | 0.02 |
|  | Haemoglobin index | -0.35 | -6.53 | 0.000 | -0.23 | -0.12 |
|  | Neu_to-Lym ratio | -0.22 | -3.25 | 0.001 | -0.07 | -0.02 |
|  | Eosinophils | 0.13 | 1.90 | 0.059 | 0.00 | 0.04 |
|  | Monocytes | -0.14 | -2.10 | 0.037 | -0.03 | 0.00 |
|  | TWBC | -0.02 | -0.28 | 0.780 | -0.03 | 0.02 |
| 4 | (Constant) |  | 0.99 | 0.323 | -0.31 | 0.93 |
|  | AGE | 0.02 | 0.45 | 0.652 | 0.00 | 0.01 |
|  | BMI | 0.05 | 0.91 | 0.365 | -0.01 | 0.02 |
|  | Haemoglobin index | -0.28 | -5.04 | 0.000 | -0.20 | -0.09 |
|  | Neu_to-Lym ratio | -0.26 | -4.04 | 0.000 | -0.08 | -0.03 |
|  | Eosinophils | 0.09 | 1.46 | 0.145 | -0.01 | 0.04 |
|  | Monocytes | -0.11 | -1.85 | 0.065 | -0.03 | 0.00 |
|  | TWBC | 0.08 | 1.23 | 0.220 | -0.01 | 0.04 |
|  | PLT | 0.01 | 0.06 | 0.949 | 0.00 | 0.00 |
|  | PDW | -0.10 | -1.82 | 0.071 | -0.04 | 0.00 |
|  | PCT | -0.22 | -2.18 | 0.030 | -2.92 | -0.15 |
|  | MPV_10000 | 0.37 | 4.09 | 0.000 | 0.03 | 0.08 |
| 5 | (Constant) |  | 1.52 | 0.131 | -0.18 | 1.34 |
|  | AGE | 0.08 | 1.17 | 0.242 | 0.00 | 0.01 |
|  | BMI | 0.02 | 0.35 | 0.729 | -0.01 | 0.02 |
|  | Haemoglobin index | -0.21 | -3.62 | **0.000** | -0.16 | -0.05 |
|  | Neu_to-Lym ratio | -0.25 | -3.92 | **0.000** | -0.08 | -0.03 |
|  | Eosinophils | 0.07 | 1.20 | 0.230 | -0.01 | 0.03 |
|  | Monocytes | -0.10 | -1.65 | 0.100 | -0.03 | 0.00 |
|  | TWBC | 0.05 | 0.75 | 0.452 | -0.02 | 0.03 |
|  | PLT | -0.10 | -0.81 | 0.421 | 0.00 | 0.00 |
|  | PDW | -0.12 | -2.24 | **0.026** | -0.04 | 0.00 |
|  | PCT | -0.13 | -1.22 | 0.224 | -2.38 | 0.56 |
|  | MPV_10000 | 0.30 | 3.17 | **0.002** | 0.02 | 0.07 |
|  | T. Bilirubin | 0.12 | 1.54 | 0.124 | 0.00 | 0.02 |
|  | Indirect Bilirubin | 0.04 | 0.63 | 0.530 | -0.01 | 0.02 |
|  | Albumin | -0.11 | -2.19 | **0.030** | -0.02 | 0.00 |
|  | AST | 0.36 | 2.16 | **0.032** | 0.00 | **0.02** |
|  | GGT | -0.12 | -2.00 | **0.047** | -0.01 | **0.00** |
|  | De_Ritis | 0.14 | 1.26 | 0.207 | -0.04 | 0.18 |
|  | APRI_INDEX | -0.42 | -1.67 | 0.097 | -1.46 | 0.12 |
|  | FIB_4 | -0.16 | -0.94 | 0.346 | -0.26 | **0.09** |
|  | RPR | 0.34 | 2.80 | **0.006** | 0.81 | 4.65 |

**Table S2 Biochemical Analytes of the Study of the study participants**

| **Serum Biochemical Analytes (RR)** | **Cases**  **(n=150)** | **Controls**  **(n=150)** | **p-value** |
| --- | --- | --- | --- |
| Total Bilirubin (µmol/l) | 14.98 ± 6.49 | 12.71 ± 5.32 | *0.001* |
| Direct Bilirubin. (µmol/l) | 6.76 ± 4.72 | 6.80 ± 4.62 | 0.94 |
| Indirect Bilirubin | 8.21 ± 6.29 | 5.91 ± 4.07 | *<0.001* |
| Total Protein (g/l) | 70.83 ± 9.94 | 72.19 ± 7.64 | 0.19 |
| Albumin (g/l) | 39.78 ± 7.32 | 41.58 ± 5.56 | 0.017 |
| ALT (U/L) | 27.73 ± 13.88 | 26.16 ± 11.77 | 0.29 |
| AST (U/L) | 30.26 ± 18.85 | 26.94 ± 13.24 | 0.08 |
| GGT (U/L) | 33.21 ± 16.85 | 29.92 ± 12.87 | 0.06 |
| ALP | 158.67 ± 53.42 | 159.28 ± 55.42 | 0.92 |
|  |  |  |  |

***Data is presented as mean ± SD.*** **** = p-values< 0.05 were considered statistically significant. RR= Reference range. ALT – Alanine transaminase, AST – Aspartate transaminase. GGT - Gamma-glutamyl transferase, ALP – Alkaline phosphate***
